# Dr. Charles M. Kellogg

**Published:** 1907-06

**Authors:** 


Dr. Charles M. Kellogg, of Rochester, N. Y., died at his home March 29, 1907, aged 61 years. He graduated at the Medical Department, University of Buffalo, in 1884.

